# Implementation of the Use of Ethnomedicinal Plants for Curing Diseases in the Indian Himalayas and Its Role in Sustainability of Livelihoods and Socioeconomic Development

**DOI:** 10.3390/ijerph18041509

**Published:** 2021-02-05

**Authors:** Munesh Kumar, Sushma Rawat, Bhuvnesh Nagar, Amit Kumar, Nazir A. Pala, Jahangeer A. Bhat, Rainer W. Bussmann, Marina Cabral-Pinto, Ripu Kunwar

**Affiliations:** 1 Department of Forestry and Natural Resources, HNB Garhwal University, Srinagar-Garhwal 249161, Uttarakhand, India; muneshmzu@yahoo.com (M.K.); rawatsushma26@gmail.com (S.R.); 2 Department of Forest Products and Utilization, College of Horticulture and Forestry, Jhalawar 326023, Rajasthan, India; bhuwi@hotmail.com; 3 School of Hydrology and Water Resources, Nanjing University of Information Science and Technology, Nanjing 210044, China; amit.agl09@gmail.com or; 4 Faculty of Forestry, Sher-e-Kashmir University of Agriculture Sciences and Technology of Kashmir, Jammu and Kashmir 190025, India; nazirpaul@gmail.com; 5 Department of Forest Products and Utilization, College of Horticulture and Forestry, Rani Lakshmi Bai Central Agricultural University, Jhansi 284003, Uttar Pradesh, India; jahan191@gmail.com; 6 Department of Ethnobotany, Institute of Botany, Ilia State University, Tbilisi 0105, Georgia; rainer.bussmann@iliauni.edu.ge; 7 Department of Geosciences, University of Aveiro, Campus de Santiago, 3810-193 Aveiro, Portugal; 8 Ethnobotanical Society of Nepal, Kathmandu 44600 Nepal; ripukunwar@gmail.com

**Keywords:** socio-economic factors, Himalayas, healthcare, rural inhabitants, medicinal plant, Forestry

## Abstract

In recent times, the use of traditional herbal medicines in healthcare has declined, particularly amongst the rural population. This implies a risk of losing vital information from previous generations regarding plants and their use in traditional medicine. The objective of this study is to catalog the ways employed by inhabitants of the Garhwal Himalayas as part of their traditional approaches to healthcare. Information was gathered through snowball sampling using a questionnaire combined with informal interviews. This was supplemented by discussions with peers and practitioners prominent in this healing technique. The homogeneity within ethnomedicinal knowledge of these rural residents was tested using the informant consensus factor (Fic). The calculation of the fidelity value (FL) and the cultural importance index (CI) were made regarding the population’s dependency on plants. A total of 88 plant species from 44 families and 80 genera were identified as medicines for various complaints. Leaves were the most frequently used plant part followed by fruits, seeds, roots, bark, and flowers/buds. The largest number of taxa (15 species) were used for treatment of skin ailments (with Fic score of 0.85) followed by wounds, coughs, and digestive problems. There was a significant relationship observed between the medicinal plants used and distance (time of access) and family income. The present study will provide baseline information to be established for future research. The available information could help to discover new drugs for the pharmaceutical industry. Thus, the study revealed that the plants that have high scores of FL and CI can be used to discover new drug extraction in the future for further studies.

## 1. Introduction

Humans have always used plants to enhance physical and spiritual wellbeing [1], and medicinal plants have continued to gain prominence, even in the modern era [2]. In the modern era, about 40,000–50,000 plant species are being used for traditional healthcare, and to discover drugs all around the globe [3]. According to the World Health Organization (WHO), 65–80% of the world’s population, particularly in developing countries, depend on plants for healing, and this is well accepted in traditional culture [4], often due to poverty, and lack of access to modern medicine [5]. Traditionally used medicinal plants have a long history of use as they are often considered safe and non-toxic to human beings [6]. Most people residing in rural areas depend on subsistence agriculture for their survival and have a great cultural knowledge of curing diseases by the utilization of forest resources [7]. However, lack of documentation of traditional practice by the healers leads to the unclear effect of herbal medicine among the present generation [8]. These traditional resources sometimes play an important role in the subsistence of local inhabitants and even income generation. Wild collection practices secure valuable income for many rural households and provide incentives for conservation and sustainable use [9]. The current market of herbal drugs is estimated at 40 billion and is expected to increase by 16% in the next 3–4 years. However, production of many herbs is less than market demand, which incentivizes adulteration in the Ayurvedic drugs [10].

Ethnobotanical knowledge arises from a complex interaction between human beings and their surrounding environment, which depends on various factors including local classification systems [11], communication through language [12], human cognition and cultural history [13], beliefs and religion [14], social networks and access to information [15]. Ethnomedicinal research is the study of unique knowledge about plant wealth and search of new resources for the preparation of herbal medicines, edible plants for consumption, and other aspects of plants [16]. In India, 20% of plant species are reported for medicinal values [17]. Uttarakhand, a Himalayan state, well known for its biotic wealth and variety of cultural heritage, covers about 12.18% of the total Indian Himalaya, and harbors more than 40% of its diverse forest types, comprising the highest cover of natural forest and alpine pastures [18]. The diverse ethnic communities (i.e., Garhwali, Jaunsari, Bhotia, Tharu, etc.) living in the state are dependent on forests for their primary healthcare and their livelihoods [7].

Herbal practice still plays a significant role in managing and curing various health problems, particularly in the remote and rural areas of India [19]. The knowledge of medicinal plant conservation and its use has developed a link between promoting environmental conservation and indigenous knowledge [20]. In the present scenario, the practice of herbal medicine has, however, been declining even in the places where it was once developed and nurtured by oral tradition down generations. The decline of herbal medicine use is especially rooted in the change of people’s attitudes towards allopathic medicine, and the wide availability of it even in small towns, although people are well aware of its possible side effects. This situation may lead to the loss of traditional and valuable information about the plants used in healthcare management in the future [21]. Allopathic medicine is, however, still out of reach for the majority of villagers. Thus, our target groups in this study were especially local poor people who have fewer facilities and live away from the cities. The documentation of traditional healthcare practices can help planners and policymakers in better management and sustainable use of such local resources.

Ethnobotany is valuable for the development of healthcare and conservation programs in different parts of the world. The documentation of ethnobotanical studies helps to preserve knowledge before traditional folklores are lost forever [22]. The World Health Organization estimates that 80% of the world’s population relies on traditional systems of medicine. These medicinal plants form an important part of the world’s economy because many modern medicines are derived from these plants. Indian indigenous systems of medicine are mainly based on the use of plants. Every year, the medicinal plant-related trade grows rapidly, and while India’s share in the global market is not very impressive (only 0.5–1%), demand for these products is increasing at an alarming rate [23]. Rural communities depend largely on herbal resources for curing diseases. This culture continues today in the form of folk medicine in different parts of the world and led to the development of traditional systems of medicine. Systematic and scientific investigations of traditional medicinal plants have also provided many valuable drugs in Western medicine [24]. Thus, considering the value of medicinal plant documentation for further conservation is highly important before they vanish from nature. The valuable plant information from this region needs to be systematically collected and documented for generations to come, apart from conserving these precious plant resources of high economic utility. The present study was designed to (i) document the use of ethnomedicinal plants in the villages of the Gharwal Himalayas (ii) to find consensus on information on the use of each plant in the study area and (iii) to understand the socioeconomic status of the people vs. the potential use of traditional medicine.

## 2. Materials and Methods

### 2.1. Study Area

**Figure 1:**
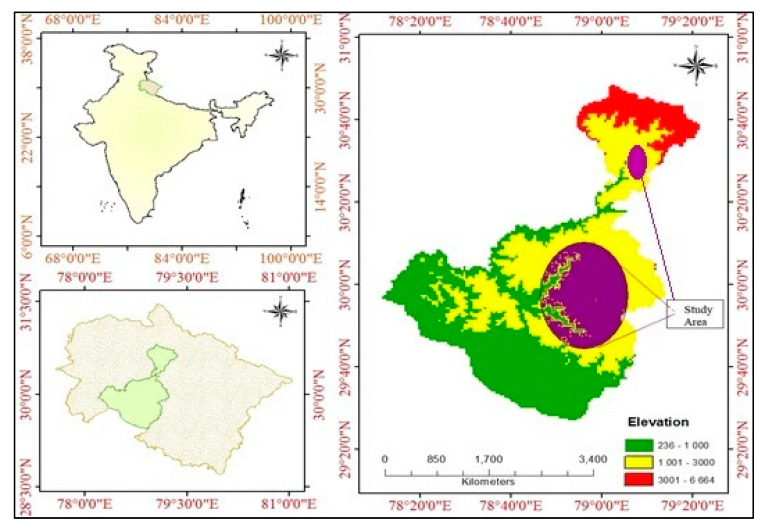
Location of the study area.

The present study was conducted in Pauri Garhwal and Rudraprayag districts of Uttarakhand, India (Figure 1). These ranges of districts extend from 29°30′–30°50′ N latitude and 78°10′–79°20′ E longitude. However, within these two districts, the different altitudinal ranges (1000–3000 m above sea level; masl) have been selected (Figure 1), and study surveyed villages were between 700 to 1800 masl. The rainfall pattern in the region is largely governed by the monsoon rains from July to September, and account for ~60–80% of the total annual rainfall [25]. The region is famous for its rich biodiversity, supporting different forest types, varying in species composition with elevation and latitude. The dominant tree species of the region reported is *Pinus roxburghii* (Chir pine).

Agriculture is the primary profession of about 80% of the people in the western and central Himalayas [26] and about 70% of them have a land-holding size of less than 1 hectare [27]. Agricultural terraces are lined with numerous trees, wild bushes, grasses, and herbs that offer inhabitants fodder for livestock [28]. Inhabitants of the study area are dependent mainly on forests for diverse needs that are critical for the maintenance of their livelihoods and wellbeing. The livelihoods of the people are directly or indirectly derived from natural resources, traditional terrace-based rainfed agriculture, and animal husbandry practices as revealed by rural inhabitants. According to the 2011 census, population of both districts is 929,546 with an average literacy rate of 82.33% (Table 1) [29]. 

### 2.2. Sampling Techniques

A test questionnaire was framed and used to test the suitability and flow of questions among 48 households with 21 and 27 in Pauri and Rudraprayag districts, respectively. After that, suitable and needful changes were made as per the requirement and incorporated in the final questionnaire (Appendix A). The final questionnaire was used to gather information through semi-structured interviews from 161 households (64 from Pauri and 97 from Rudraprayag). The snowball sampling technique was used for the selection of informants that had a sound traditional knowledge of medicinal plants used in the area. In the beginning, we approached the “Gram Pradhan” (a representative of the village; who keeps all information of the villages, including documentary proof) and older people of the village, who were reputed to know the medicinal plants used in the treatment of various health ailments. Once a traditional healer/plant collector was identified, snowball sampling was followed to locate and identify other respondents [30]. Since the younger generation had less awareness about traditional knowledge, respondents in the age group of 50–80 years were interviewed. A large number of respondents (75.3%) were literate and friendly in disclosing the information about the traditional medicines that were passed on to them from their ancestors. The respondents of selected households were interviewed in their local language, i.e., Garhwali or Hindi. Before starting an interview, the inhabitants were advised about the purpose of the study and interview. Most of the information about the medicinal plants was recorded from the older people of the villages, including the importance of medicinal plants, plant parts used, name of the disease for which a particular plant was being used, etc. The published literature and consensus discussions with the inhabitants were also used for comparison. The used plants were collected and identified and confirmed from the traditional healers and participants. The Flora of Garhwal Himalayas was used to crosscheck the species local name and scientific nomenclature [31,32]. The specimens were then processed in the laboratory, verified by the curator, and submitted to Garhwal University Herbarium (GUH).

### 2.3. Data Analysis

The data collected during the fieldwork were analyzed for various parameters, i.e., informant’s consensus factor (Fic), fidelity value (FL%), and cultural importance index (CI). A consensus survey was conducted based on people’s agreement on the number of plants used for a particular health ailment. To test the homogeneity of traditional medicinal knowledge about the plants, the informant’s consensus factor (Fic) was used [33,34]. The Fic for each of the recorded plant species was calculated using the following formula:

(1) $$F_{\mathrm{ic}}=\frac{N_{\mathrm{ur}}-N_{t}}{N_{\mathrm{ur}}-1}$$

where N_ur_ is the number of use reports for a particular health problem and N_t_ is the number of species used for a particular health problem by all the informants. The resulting factor ranges from 0 to 1, where high value reveals high rate of informant consensus. The fidelity level [35] is the percentage of informants claiming the use of a certain plant species for the same major purpose, and was calculated as:

(2) $$\mathrm{Fidelity}\;\mathrm{value}\;\left(\mathrm{FL},\;\%\right)=\frac{I_{p}}{I_{u}}\times 100$$

where I_p_ is the number of informants indicating independent use of a species for the same major ailment and I_u_ is the total number of informants mentioning the use of plants for any major ailment. Cultural Importance Index (CI) was calculated by dividing the number of use report (UR) in use-category by the number of informants [33] to assess the importance of each species using the following formula:

(3) $${\mathrm{CI}}_{s}=\;\overset{u_{\mathrm{NC}}}{\underset{u=u_{1}}{\sum }}\overset{i_{N}}{\underset{i=i_{1}}{\sum }}\frac{{\mathrm{UR}}_{\mathrm{ui}}}{N}$$

where UR is the number of useful reports in various health problems (NC) and (N) is the total number of informants.

One-way ANOVA and binary logistic regression analysis were used to estimate the relationship between various household factors and the use of medicinal plants. The description of various explanatory variables has been provided in Table 2, where resources for each category and indicator selected for the household environment were aggregated through relevant indices after normalizing each sub-category, respectively, through the standard protocol of min-max approach. The statistical test of significance of variables is given in Table 3.

## 3. Results

**Figure 2:**
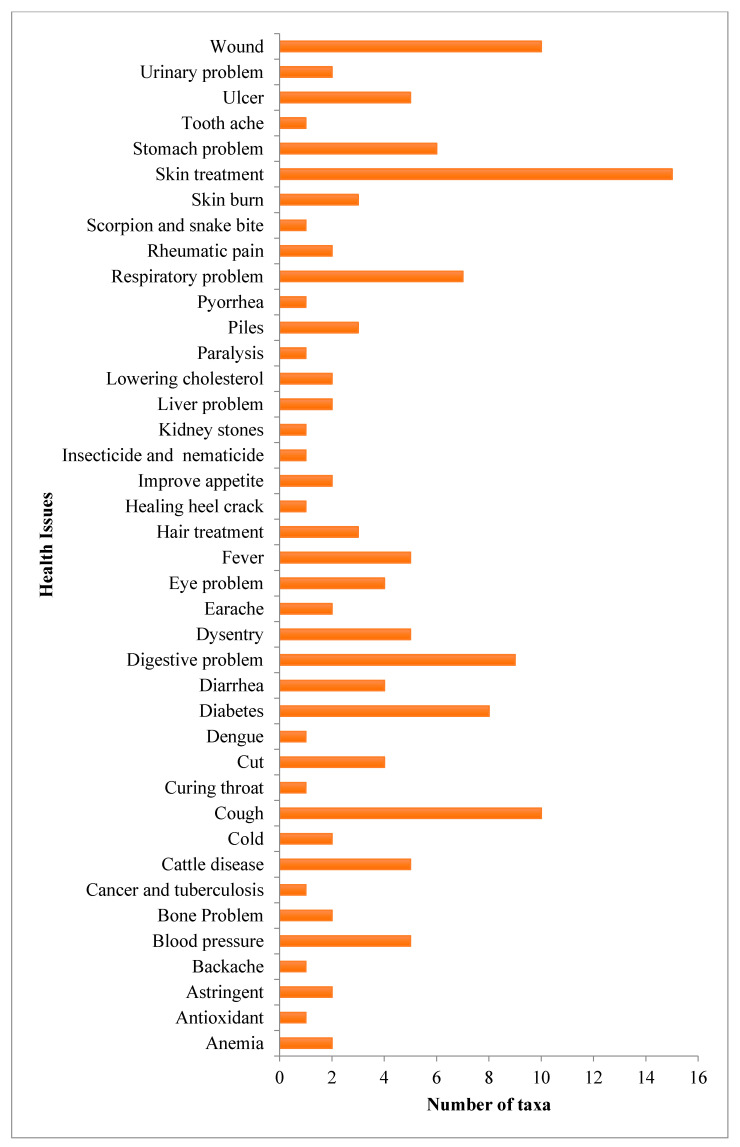
Number of plant species (taxa) used to treat different health conditions.

The existing traditions of managing different diseases by the inhabitants of Pauri and Rudraprayag district were recorded and the complete details are presented in Appendix B. A total of 88 medicinal plants were recorded from 44 families and 80 genera. The plant parts were used including leaves, roots, fruits, seeds, bark, flowers, and underground parts. In the present study, the maximum number of plant species (15 species) were used for skin treatment followed by the wound, cough (10 each); digestive problems (09); diabetes (08); respiratory problems (07); stomach problems (06); blood pressure, cattle diseases, dysentery, fever, ulcer (05); cut wounds, diarrhea, eye problems (04); piles, hair treatment, skin burn (03 each); rheumatic pain, cholesterol problems, liver problems, appetite improvement, earache, cold, bone problems, astringent, anemia, urinary issues (02 each); antioxidant, backache, cancer and tuberculosis, throat infections, dengue, heel crack, insecticide and nematicide, kidney stones, paralysis, pyorrhea, scorpion bites and snake bites, toothache (01 each) (Figure 2).

**Figure 3:**
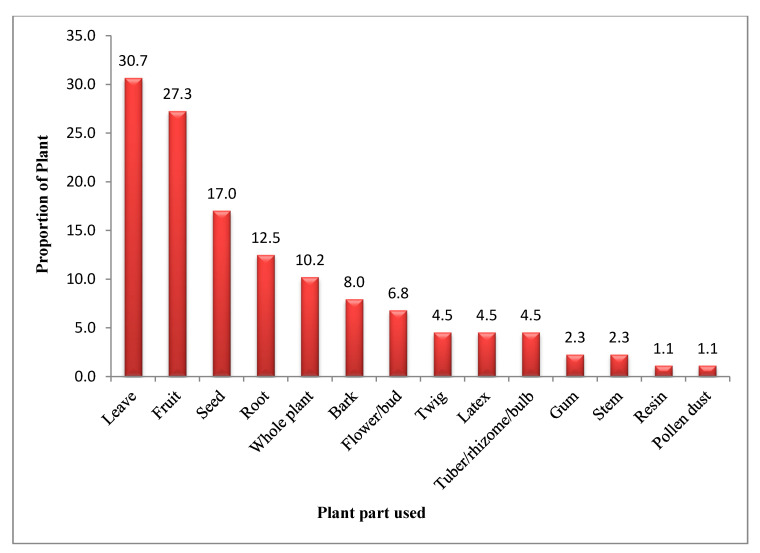
Proportion of different plant parts used in curing health problems.

Among the different plant parts used to cure various diseases, leaves contributed the most (30.7%), followed by fruits (27.3%), seeds (17%), roots (12.5%), bark (10.2%), flowers (8%), whole plants (6.8%); tuber/rhizome/bulb, twig, latex (4.5% each); gum and stem (2.3% each) and resin, pollen dust (1.1% each) (Figure 3). Practice of using medicinal plant resources in healthcare management by rural households’ as a part of their cultural tradition is passed on to them from generations. This observation during the study reveals that local people are dependent on these plants for their daily needs, especially medicines and nutrition.

### 3.1. Health Problem and Uses 

Medicinal plants used for different ailments were classified into 40 groups and Fic values for each category are mentioned in Table 4. The results of the Fic showed that the antioxidant; back-ache; cancer and tuberculosis (pollen dust and resin of *Pinus roxburghii* Sarg. with water is useful for tuberculosis); dengue, healing heal crack; insecticide and nematicide; kidney stones; paralysis; pyorrhea; scorpion and snake bites; toothache and throat infections; category had the greatest agreement with a Fic of (1.00), followed by cold, appetite improvements and cholesterol level reduction (0.98); hair treatments (0.97); urinary infections and bone problems (0.96); earache and eye problems (0.95); astringent and cuts (0.94). The least agreement between the informants was recorded in the piles and ulcer with Fic value of (0.50). Skin problems were cured with the highest number of taxa (15 spp.) with Fic value of 0.85 (Table 3). The awareness about the species used in skin-related problems was observed as high in the study area. 

The inhabitants of the study area are engaged in various activities and have to face many issues while collecting fuelwood and fodder; cooking food in traditional stoves; agricultural farming on hilly terrains; carrying portable water from distant places. These situations lead inhabitants to suffer from many skin-related problems, such as ringworm, skin disorders, skin allergy, fungal infection, skin dryness, skin infection, etc.

### 3.2. Cultural Importance of Medicinal Plants

The study results revealed that *Trigonella foenum-graecum* and *Allium sativum* with CI value 0.292 were the most used species in healthcare management with 47 use reports each. The results further reported that the most important species used in treating skin-related problems were *Artemisia wallichiana* and *Phyllanthus emblica* with CI value 0.273 and 0.205 respectively indicating more use of these species by the inhabitants due to the availability of plant species and knowhow of their use in treating skin problems. The ANOVA analysis shows that distance of household from the hospital (F = 3.600; *p* < 0.05) and monthly income of the family (F = 8.614; *p* < 0.05) are the main influencing factors favoring the use of medicinal plants for curing health problems, followed by a distance of household from forest (F = 4.766; *p* < 0.05) (Table 2b).

## 4. Discussion

The results revealed that local traditional knowledge plays an essential role in primary healthcare and the practice of plant-based medicine is still prevalent in rural areas of the Garhwal region. Traditional herbal medicine is considered as the lifeline, the first choice, with fewer side effects, better patient tolerance, relatively economic, cultural recognition, and long history of use, in comparison to pharmaceutical medicine [36]. Local people show preferences for the use of traditional herbal remedies due to their belief in the effectiveness of folklore herbal remedies [19]. In this study, it was found that the use of ethnomedicinal plants was higher at increasing elevation due to a lack of alternative options and accessibility to markets. The use of indigenous medicine through local healers in remote areas is more demanding due to low prices because the cost of modern medicine is very high [37].

In an ethnomedicinal study of Kedarnath Wildlife Sanctuary, Malik et al. [19] reported that the most used plant part was roots (33%), followed by leaves (27%), bark (20%), etc., which is different from our findings as the inhabitants were less aware of the medicinal use of root parts. Sharma et al. [25] also reported uses of different plant parts and the most commonly used plant part in the preparation of herbal ointments was leaves followed by seeds, roots, whole plant, stem, flower, and fruit. Ayyanar and Ignacimuthu [38] suggested that most of the studies confirmed the medicinal uses of leaves in the treatment of various health illnesses.

Informant’s consensus factor is used to evaluate the reliability of the informant’s information about plant use [39]. High Fic value indicates the use of some plants by many inhabitants in curing a particular health illness whereas low value means the use of different plants by many inhabitants in curing a particular health illness [40]. In the present study, informant’s consensus survey indicated that twelve (12) plant species were most commonly used for individual diseases, and therefore the informant’s consensus index factor was high (1.00). However, the lowest informant’s consensus (0.50) was reported for piles and ulcers where only 3 and 5 plants were used for the curing diseases, respectively. The high degree of consensus of the informants indicated that current use and knowledge are still strong, so preservation of existing traditional knowledge needs to be done before much has been lost [39]. Therefore, local people showed high agreement on the usages of plants for specific ailments from the present study. The cultural importance of a plant depends on the versatility of plants with different uses to those with only one use [33]. This study also claimed the use of Artemisia spp. in treating various skin diseases [41].

Medicinal plants and their traditional formulations have always been a part of social life in rural communities, which have proved to be very helpful in tackling various health-related issues [42]. The dependency of the villagers on medicinal plants increased due to a lack of other healthcare facilities close to their households. Thus, informants of the study area reported that medicinal plants served as an important source for their healthcare, and the associated knowledge, which was traditionally transmitted and thus improved health conditions of human beings [43], and older participants clearly had a preference for the user to the ethnomedicinal plants for curing different health issues. The wider application and adaptation of uses of ethnomedicinal plants and the inclusion of traditional knowledge in decision-making processes at highest level are of great importance. Most of the documented species in the present study have also been reported for multiple uses in various regions of the globe (Table 5). Multiple uses of these plants may incite the appropriate authorities to frame better conservation and management strategies for the plants used for medicinal purposes.

Pharmaceutical medicines cure a range of diseases; however, their higher prices and side effects limit their applications. It is also observed that those living in remote areas who associated with nature used medicinal plants from generations to coming generation although they have minimum side effects, no doubt due to many cases of poisoning associated with herbal medicines increasing in different parts of the world. Therefore, it is also necessary to ensure toxicity assessment on these products for safe use to protect health [38]. In the recent decades, rapid changes in urbanization and its influence on cultural settings has led to the depletion of traditional knowledge in several areas including the Himalayas, due to unorganized way of knowledge transfer to the new generations [44]. This state of affairs can lead to the eradication of vast ethnomedicinal understanding of the region if proper documentation is not taken care of. Therefore, to preserve traditional medicinal knowledge, the importance of herbal practitioners and their role in primary healthcare systems should be recognized at the regional as well as national level. This can be achieved by the capacity building of herbal practitioners and education of new generations, which will have a substantial impact on the long-standing sustainability of herbal knowledge [45]. Furthermore, there is an urgent need to document this information, as it is rapidly declining due to the influence of pharmaceutical medicines [46]. Thus, it is important to collect this information and develop a database of medicinal plants for future research and potential development of new herbal medicines.

Uttarakhand has great potential for the cultivation of medicinal plants as important sources of sustainable livelihoods. Medicinal and aromatic plants can play an important role in the subsistence livelihood enhancement of rural people. In Uttarakhand State, MAP (Medicinal and Aromatic plants) are an important non-timber forest product and their sustainable trade and commercialization have the potential to generate employment and improve the economy of many rural communities. The region’s diverse geo-climatic conditions and rich availability of forest-grown MAP resources mean it has much potential to become a vibrant hub for Northern India’s MAP industry. However, current management practices including a disorganized pattern of trade are hampering the conservation of Uttarakhand MAP resources and their potential for sustainable commercialization. The remoteness of many communities in the region is restricting their participation in the industry. There are also limited data on the quantity of MAP being supplied from the region, and so their monetary contribution to India’s overall MAP trade is not well defined. To help overcome the challenges facing the MAP industry in Uttarakhand, there is a need to better understand the sector’s complex socioecological and socioeconomic conditions and interrelationships. Although there is currently trade and commercialization of MAP in the region, it is not considered to be working within a sustainable business platform. Hence, there is a need to find practical solutions for the sustainable commercialization of MAP in Uttarakhand State. This requires developing strategic marketing prospects for individual businesses and the industry as a whole.

## 5. Conclusions

This study provides broad information about the traditional knowledge of medicinal plants used by rural inhabitants of Garhwal Himalaya, which is under threat of being lost in the near future because of climate change vulnerability in the Himalaya region. Medicinal plants and their traditional formulations have always been a part of the livelihood of rural inhabitants, which have proved to be very useful in dealing with various health issues. Results of this study showed a significant relation between medicinal plant use with a distance of hospital from the household, monthly income of family, and distance of forest from household. Thus, it can be concluded that rural inhabitants of Garhwal Himalaya have sound ethnomedicinal knowledge of curing various health-related issues by the use of local medicinal plants accessible to them from their vicinity. 

This study provides baseline information for more scientific studies that may lead to the discovery of new plant-based drugs that will help in the development of effective herbal medicines in the coming decades. In the present situation, more dependency of young people on allopathic medicine systems has led to the degradation of traditional knowledge systems. Therefore, the present circumstances must document the traditional knowledge related to medicinal uses of plants and their conservation for future generations. This is important because currently, the younger generation is barely interested in learning and using old age practices. The findings of the study highlight a need for the development of methods or policies which can help in conserving the traditional knowledge of plants used by rural inhabitants in healthcare and thus, in sustaining rural health problems.

## Figures and Tables

**Table 1:** Demographic status of the study area (Source: Census of India, 2011).

Parameter	Uttarakhand	District	
		Pauri Garhwal	Rudraprayag
Population			
Male	5,137,773	326,829	114,589
Female	4,948,519	360,442	127,696
Literacy rate (%)			
Male	87.4	92.71	93.90
Female	70.01	72.60	70.35
Number of villages			
Inhabited villages	15,745	3142	653
Uninhabited villages	1048	331	35

**Table 2:** Description of variables used.

Variable Name	Description
GEN	Gender of respondent (1 if male, 0 for female)
AGE	Age of respondent (in years)
EDU	Education level of respondent (0 for illiterate, 1 for literate, 2 for primary, 3 for high school, 4 for intermediate, 5 for bachelor and above
PROF	Profession (1 for others, 2 for wage labor, 3 for business, 4 for agriculture, 5 for service)
HEAL	Healer (1 for yes, 0 for no)
DISTFOR	Distance from forest (in km)
DISTHOSP	Distance from hospital (in km)
INCOME	Monthly income (1 for Below, 2 for Rs. 3000–6000, 3 for Rs. 6000–12,000, 4 for Rs. 12,000–24,000)

**Table 3:** ANOVA test for significance of variables.

Variables	Average	Std Dev	Standard Error (SE)	F Value (sig.)
GEN	-	-	-	Ns
AGE	62.211	8.6627	0.6827	Ns
EDU	-	-	-	Ns
PROF	-	-	-	Ns
HEAL	-	-	-	Ns
DISTFOR	1.646	0.9281	0.0731	4.766 (0.001)
DISTHOSP	8.646	6.7446	0.5315	3.600 (0.000)
INCOME	-	-	-	8.614 (0.000)

Ns: non-significant.

**Table 4:** Informant consensus of ethnomedicinal plants.

Health Problems (*F_ic_*)	Number of Taxa Used (*N_t_*)	Number of Use Reports (*N_ur_*)	Species Fidelity Value (*FL%*)
Anemia (0.93)	2	15	*Morus serrata* (100%), *Luffa cylindrica* (46.2%)
Antioxidant (1.00)	1	12	*Prunus armeniaca* (42.9%)
Astringent (0.94)	2	18	*Prunus cerasoides* (28.2%), *Ziziphus nummularia* (77.8%)
Backache (1.00)	1	21	*Prunus cerasoides* (53.8%)
Blood pressure (0.92)	5	54	*Musa paradisiaca* (73.7%), *Bauhinia variegata* (54.5%), *Carica papaya* (42.9%), *Cleome viscose* (100%), *Rhododendron arboreum* (100%)
Bone Problem (0.96)	2	28	*Sesamum orientale* (100%), *Bombax ceiba* (100%)
Cancer and tuberculosis (1.00)	1	2	*Pinus roxburghii* (5.1%)
Cattle disease (0.90)	5	43	*Prunus persica* (100%), *Quercus leucotrichophora* (100%), *Juglans regia* (66.7%), *Reinwardita indica* (100%), *Punica granatum* (100%)
Cold (0.98)	2	56	*Adhathoda vasica* (60%), *Allium sativum* (100%)
Cough (0.89)	10	85	*Datura stramonium* (60%), *Myrica esculenta* (33.3%), *Psidium guajava* (100%), *Ocimum sanctum* (85.7%), *Cinnamom tamala* (23.1%), *Terminalia chebula* (65.2%), *Terminalia belerica* (87.5%), *Adhathoda vasica* (20%), *Mangifera indica* (48.3%), *Semecarpus anacardium* (33.3%)
Curing throat (1.00)	1	15	*Cinnamomum tamala* (57.7%)
Cut (0.94)	4	52	*Ficus roxburghii* (37.1%), *Rhus parviflora* (35.0%), *Anaphalis adnata* (66.7%), *Eupatorium adenophorum* (64.9%)
Dengue (1.00)	1	5	*Carrica papaya* (15.2%)
Diabetes (0.78)	8	33	*Aegle marmelos* (100%), *Ficus subincisa* (33.3%), *Syzizium cumini* (100%), *Musa paradisiaca* (15.8%), *Ocimum sanctum* (14.3%), *Berberis asiatica* (36.4%), *Momordica charantia* (100%), *Asparagus curillus* (100%)
Diarrhea (0.73)	4	12	*Citrus limon* (100%), *Ficus subincisa* (33.3%), *Ficus roxburghii* (11.4%), *Semecarpus anacardium* (8.3%)
Digestive problem (0.87)	9	64	*Prunus cerasoides* (17.9%), *Myrica esculenta* (66.7%), *Zea mays* (100%), *Ziziphus mauritiana* (100%), *Oogenia oojenensis* (100%), *Tinospora cordifolia* (12.9%), *Raphanus sativus* (52.4%), *Lagenaria siceraria* (31%), *Mangifera indica* (51.7%)
Dysentery (0.85)	5	27	*Ficus roxburghii* (25.7%), *Echinochloa crus-galli* (100%), *Butea monosperma* (33.3%), *Woodfordia fruticosa* (100%), *Terminalia belerica* (12.5%)
Earache (0.95)	2	23	*Perilla frutescens* (100%), *Artemisia wallichiana* (36.4%)
Eye problem (0.95)	4	61	*Hordeum vulgare* (100%), *Pyrus pashia* (100%), *Glycine max* (100%), *Berberis asiatica* (63.6%)
Fever (0.91)	5	47	*Cynodon dactylon* (38.9%), *Vitex negundo* (19%), *Tinospora cordifolia* (71%), *Cannabis sativa* (100%), *Carissa carandus* (37.5%)
Hair treatment (0.97)	3	67	*Grewia optiva* (100%), *Phyllanthus emblica* (69.7%), *Allium cepa* (100%)
Healing heel crack (1.00)	1	37	*Pinus roxburghii* (94.9%)
Improve appetite (0.98)	2	65	*Brassica campestris* (100%), *Amaranthus spinosus* (74.2%)
Insecticide and nematicide (1.00)	1	3	*Ageratum coyzoides* (100%)
Kidney stones (1.00)	1	26	*Macrotyloma uniflorum* (100%)
Liver problem (0.90)	2	11	*Raphnus sativus* (19%), *Luffa cylindrica* (53.8%)
Lowering cholesterol (0.98)	2	63	*Prunus armeniaca* (57.1%), *Trigonella foenumgraecum* (100%)
Paralysis (1.00)	1	7	*Vitex negundo* (33.3%)
Piles (0.50)	3	5	*Ficus subincisa* (33.3%), *Terminalia chebula* (4.3%), *Dioscorea bulbifera* (66.7%)
Pyorrhea (1.00)	1	11	*Bauhinia vahlii* (100%)
Respiratory problem (0.80)	7	31	*Murraya koenigii* (100%), *Datura stramonium* (26.7%) (13.3%), *Musa paradisiaca* (10.5%), *Cinnamom tamala* (19.2%), *Tinospora cordifolia* (16.1%), *Terminalia chebula* (26.1%) (4.3%), *Adhathoda vasica* (13.3%) (6.7%)
Rheumatic pain (0.80)	2	6	*Abrus precatorius* (66.7%), *Vitex negundo* (19%)
Scorpion and snake bite (1.00)	1	2	*Achyranthes aspera* (100%)
Skin burn (0.94)	3	37	*Curcuma angustifolia* (100%), *Cynodon dactylon* (61.1%), *Sapium insigne* (100%)
Skin treatment (0.85)	15	93	*Rosa rubiginosa* (100%), *Rubus ellipticus* (60%), *Rumex hastatus* (100%), *Ziziphus nummularia* (22.2%), *Butea monosperma* (66.7%), *Juglans regia* (33.3%), *Melia azederach* (100%), *Raphanus sativus* (28.6%), *Bauhinia variegata* (45.5%), *Shorea robusta* (100%), *Phyllanthus emblica* (30.3%), *Semecarpus anacardium* (58.3%), *Coriandrum sativum* (100%), *Calotropis procera* (100%), *Artemisia wallichiana* (27.3%) (20.5%)
Stomach problem (0.91)	6	59	*Urtica dioica* (100%), *Oryza sativa* (100%), *Mentha arvensis* (100%), *Carica papaya* (57.1%), *Lagenaria siceraria* (31%), *Amaranthus spinosus* (25.8%)
Tooth ache (1.00)	1	23	*Zanthoxylum armatum* (100%)
Ulcer (0.50)	5	9	*Rubus ellipticus* (40%), *Oxalis corniculata* (30%), *Abrus precatorius* (33.3%), *Dioscorea bulbifera* (33.3%), *Eupatorium adenophorum* (5.4%)
Urinary problem (0.96)	2	27	*Lagenaria siceraria* (37.9%), *Cucumis sativus* (100%)
Wound (0.88)	10	76	*Ficus roxburghii* (25.7%), *Ficus palmata* (100%), *Oxalis corniculata* (70%), *Vitex negundo* (28.6%), *Rhus parviflora* (65%), *Carissa carandus* (62.5%), *Anaphalis adnata* (33.3%), *Tagetus minuta* (100%), *Eupatorium adenophorum* (29.7%), *Artemisia wallichiana* (15.9%)

**Table 5:** Existing literature of plant species used in different ailments.

Plant Species	Ailments
*Justicia adhatoda*	Chronic cold and cough, piles, leprosy, and diabetes [47]; cough and cold, chronic bronchitis [48]
*Achyranthes aspera*	Anti-amoebic and anti-fertility activity [49]; treatment of cancer, leprosy, asthma, fistula, piles, arthritis, wound, insect and snake bite, dandruff, hepatitis [50]
*Allium cepa*	Edible condiments, vegetable [51]
*Allium sativum*	Cardiovascular disease, diabetes, blood pressure [52]
*Phyllanthus emblica*	Constipation, fever, itching, digestive [53,54,55]
*Coriandrum sativum*	Antioxidant [56]
*Calotropis procera*	Antitumor, antihelmintic, antioxidant [57]
*Carissa carandas*	Anti-inflammatory and anti-pyretic activity [58]
*Asparagus curillus*	Piles, fever, wound, anti-toxic, weakness, cough [53]; epilepsy [59]
*Mangifera indica*	Cough and cold, dysentery, worm, furniture, leaf religious [53]
*Ageratum conyzoides*	Muscular pain, piles, ring worm, snake bite [60]; control bleeding [61]
*Anaphalis adnata*	Juice applied on fresh cuts and wounds [62]
*Artemisia vulgaris*	Nervous and spasmodic affections, asthma [63]
*Bauhinia vahlii*	Dysentery [64]
*Cannabis sativa*	Diarrhea and body pain [64]
*Eupatorium adenophorum*	Juice applied on fresh cut [64]
*Berberis asiatica*	Conjunctivitis and eye inflammation [65]
*Brassica campestris*	Fever, indigestion, and irritation [66]
*Oxalis corniculata*	Diarrhea, piles, anemia, and eye problems [62,66]
*Mentha arvensis*	Rheumatism, fever, weakness, ulcer, wounds, jaundice, cough, asthma, and cuts [66]
*Dioscorea bulbifera*	Edible [67]
*Carica papaya*	Heart problem, skin problem, piles [53]; bone fracture [67]
*Terminalia belerica*	Fruit for piles, dropsy, diarrhea, leprosy, headache [68]; cold, constipation, piles [60]
*Terminalia chebula*	Digestion, skin problem [53]; cold, cough, fever, stomach ache [69]; diabetes [70]
*Lagenaria siceraria*	Jaundice, diarrhoea, and dysentery [71,72]
*Melia azedarach*	Stomatitis, internal worm, stone in urinary bladder [53]; fever [72]
*Murraya koenigii*	Anemia, vomiting, wound [53]; vomiting, dysentery [72]
*Ocimum sanctum*	bronchitis, asthma, and genitourinary disorders [73]; cold and cough [48]
*Perilla frutescens*	Cough and nausea [74]
*Cinnamon tamala*	Antigonorrhoeic, hypoglycemic, stimulant, anti-rheumatic, and antidote for scorpion sting [75,76]
*Abrus precatorius*	Antidote, dental caries, baldness, dandruff, erysipelas [77]
*Butea monosperma*	Tuberculosis [78]
*Syzizium cumini*	Dysentery [78]
*Glycine max*	Cholesterol lowering and anticancer [79]
*Trigonellafoenum-graecum*	Diabetes, stomach complaints [80]; easier delivery [81]
*Punica granatum*	Diarrhea, fever, indigestion [82]; heart problem, eye, and ear disorder, jaundice [53]
*Woodfordia fruticosa*	Leprosy, toothache, leucorrhea, fever, dysentery, bowel disease [83]
*Ficus subincisa*	Boils [84]
*Datura stramonium*	Against rabies, nervousness, nausea, and hysteria [84]
*Myrica esculenta*	Sinusitis [85]
*Sesamum orientale*	Skin for sunburns and ringworm [86]
*Cynodon dactylon*	Antiseptic, snake bite, stop bleeding, wounds, miscarriage [87,59]
*Hordeum vulgare*	Beriberi, cough, influenza, measles, syphilis, nephritis, jaundice, dysentery, abortion, common cold, kidney diseases, skin diseases [88]
*Tinospora cordifolia*	Piles, eye problem, fever, jaundice [53]; jaundice [72]
*Ziziphus mauritiana*	Dried fruits use for anodyne, anticancer, refrigerant, sedative, stomachache [89]
*Aegle marmelos*	Stomach-ache, cures cough, good for asthma, tumors [90]; dysentery [80]
*Grewia optiva*	Antibacterial, antifungal, antioxidant [91]
*Rhododendron arboreum*	Diarrhea and headache [66]
*Rhus parviflora*	Antimicrobial [54]
*Semecarpus anacardium*	Anti-atherogenic, anti-inflammatory, antioxidant, antimicrobial, anti-reproductive [55]
*Shorea robusta*	Dysentery, antidote [53]; burning sensation, chest pain, small pox [60]
*Vitex negundo*	Headache, stomach problem, diarrhea, rheumatism, bone fracture, body swelling, swelling of joints, cancer, liver complaints, jaundice, fuelwood branches for making baskets [61,69,92]

## Data Availability

Data sets used in this study is available on reasonable request from corresponding or first author.

## Abbreviations

Fic	informant consensus factor
FL	fidelity value
CI	cultural importance index
WHO	World Health Organization
GUH	Garhwal University Herbarium
NC	health problems
N	total number of informants
N_t_	Number of taxa used
N_ur_	Number of use reports
MAP	Medicinal and Aromatic plants
NS	non-significant
GEN	Gender of respondent
AGE	Age of respondent
EDU	Education level of respondent
PROF	Profession
HEAL	Healer
DISTFOR	Distance from forest
DISTHOSP	Distance from hospital
INCOME	Monthly income

## Appendix A

Questionnaire was used to collect information on plant use.	
Informant Details	
Name:	
Sex:	
Age:	
Village name:	Panchayat name:
Block name:	District name:
Main occupation:	Subsidiary occupation:
Education:	Source income/Monthly income:
Ethnobotanical uses of plants:	
1. Local/vernacular name of plant:	
2. Scientific name of plant:	
3. Part used of plant:	
4. Name of ailment/other purposes in which plant part is used:	
5. Mode of preparation:	
6. Use (externally/internally):	
7. Availability in natural habitat:	
8. Cause of declining of ethnobotanical plants if any (overgrazing, encroachments, forest fire, mining activities, climatic change, and others):	
9. Who knows best about plant and uses: vaids, shepherds, old people/new generation, and others:	
10. Any ethnobotanical plant species under cultivation:	
11. Any awareness camps/trainings/exposure visits organized for ethnobotanical plants:	
12. Any conservation practices on ethnobotanical plants:	

## Appendix B. Medicinal Plants Used for Healthcare Practices by Inhabitants

**Scientific Name and Family**	**Voucher Specimen No.**	**Local Name**	**Use Reports**	**Habit**	**Part Used**	**Medicinal Use**	**Other Uses**	**Occurrence Status** **(Gaur, 1999)**	**CI**
Acanthaceae									
*Adhatoda vasica* Nees	GUH-21001	Basingu	15	Shrub	Flower	Flower with honey is used for treating bronchitis, asthma, cough and cold.	Leaves as livestock fodder	Fairly common	0.093
Amaranthaceae									
*Achyranthes aspera* L.	GUH-21002	Latjeera	2	Herb	Whole plant	Paste of whole plant is used for treating scorpion and snake bite.	None	Fairly common	0.012
*Amaranthus spinosus* L.	GUH-21003	Marsu	31	Herb	Leaf, Seed	Helpful in improving appetite, constipation.	Nutrient supplement	Common	0.193
Amaryllidaceae									
*Allium cepa* L.	GUH-21004	Pyaaj	17	Herb	Bulb	Juice extracted from bulb is used in lice treatment	Whole plant is used as nutrient supplement	Common	0.106
*Allium sativum* L.	GUH-21005	Lahsun	47	Herb	Bulb	Bulb heated with mustard oil is helpful in treating cold.	Whole plant is used as flavouring agent in Indian dishes	Common	0.292
Anacardiaceae									
*Mangifera indica* L.	GUH-21006	Aam	29	Tree	Fruit, Leaf, Twig	Leaves are used for cough treatment and fruit is helpful in digestion.	Nutrient supplement, ritual, fuelwood	Abundant	0.18
*Rhus parviflora* Roxb.	GUH-21007	Tunglu	20	Shrub	Leaf	Leaf paste is applied to wound or cut to stop bleeding.	Leaves are used for livestock fodder and twig for tooth cleaning	Abundant	0.124
*Semecarpus anacardium* L.	GUH-21008	Bhilow	12	Tree	Fruit	Helpful in treating skin allergy, cough, diarrhea.	None	Fairly common	0.075
Apiaceae									
*Coriandrum sativum* L.	GUH-21009	Dhaniya	8	Herb	Leaf, Seed	Leaf paste is applied on skin disease.	Leaf and fruit are used as condiment.	Commonly cultivated	0.050
Apocyanaceae									
*Calotropis procera* (Aiton) W.T. Aiton	GUH-21010	Aak	5	Shrub	Root, Latex	Helpful in treating skin problems.	None	Common	0.031
*Carissa carandas* L.	GUH-21011	Karonda	8	Shrub	Root, Fruit	Crushed root is used to cure wound. Fruit is helpful in fever treatment.	Fruit is edible	Often cultivated	0.05
Asparagaceae									
*Asparagus curillus* Buch.- Ham. ex Roxb.	GUH-21012	Satavar	2	Herb	Root	Powdered root is used to cure diabetes.	None	Common	0.012
Asteraceae									
*Ageratum conyzoides* L.	GUH-21013	Janglipudina	3	Herb	Whole plant	Used as insecticide and nematicide.	None	Common	0.019
*Anaphalis adnata* Wall. ex. DC.	GUH-21014	Bugulu	12	Herb	Leaf	For healing cuts and wounds.	None	Common	0.075
*Artemisia vulgaris* L.	GUH-21015	Kunju	44	Herb	Leaf	Leaf paste is useful for skin infection, ringworm and wound. Leaf juice is used for earache.	For ritual purpose	Fairly common	0.273
*Eupatorium adenophorum* Spreng.	GUH-21016	Basya	37	Shrub	Leaf	Paste of leave paste is applied on cuts and wounds. Paste with mustard oil is used for ulcer treatment.	Used as firewood	Common	0.230
*Tagetus erecta* L.	GUH-21017	Gainda	11	Herb	Flower	Curing skin wound	For ritual purpose	Common	0.068
Berberidaceae									
*Berberis asiatica* Roxb.	GUH-21018	Kingora	33	Shrub	Root	Root after soaking in water is used for diabetes treatment and root juice is used to cure conjunctivitis	Nutrient supplement	Fairly common	0.205
Brassicaceae									
*Brassica campestris* L.	GUH-21019	Sarso	42	Herb	Leaf, Seed	For treating poor appetite	Nutrient supplement and flavoring agent in Indian dishes	Often cultivated	0.261
*Raphanus sativus* L.	GUH-21020	Muli	21	Herb	Whole plant	Helpful in curing jaundice, skin disorders and digestive problems	Nutrient supplement, salad	Commonly cultivated	0.130
Cannabaceae									
*Cannabis sativa* L.	GUH-21021	Bhang	11		Leaf, Seed	Leave or seed with pepper, cumin seeds is used for treating fever.	Seed as nutrient supplement.	Common	0.068
Caricaceae									
*Carica papaya* L.	GUH-21022	Papita	33	Tree	Seed, Fruit, Leaf	In treating blood pressure, constipation and dengue.	Nutrient supplement	Widely cultivated	0.205
Cleomaceae									
*Cleome viscosa* L.	GUH-21023	Jakhiya	17	Herb	Seed	Useful in treating high blood pressure.	Used as condiment in dishes	Common	0.106
Combretaceae									
*Terminalia belerica* Roxb.	GUH-21024	Bahera	8	Tree	Fruit	Fruit rind is used for treating cough and dysentery. Used in triphala.	Nutrient supplement	Common	0.05
*Terminalia chebula* Retz.	GUH-21025	Harad	23	Tree	Fruit	Fresh or boiled fruit pulp with honey in treating of asthma, cough and bronchitis. Also used in curing piles.	For livestock fodder	Common	0.143
Cucurbitaceae									
*Cucumis sativas* L.	GUH-21026	Kakdi	16	Climber	Seed	Seed paste mixed with water is useful in urinary problem.	Fruit as nutrient supplement	Commonly cultivated	0.099
*Lagenaria siceraria* (Molina) Standl.	GUH-21027	Lauki	29	Climber	Fruit	Helps in curing urinary disorders, indigestion and stomach acidity	Nutrient supplement	Cultivated	0.18
*Luffa cylindrica* (L.) M. Roem.	GUH-21028	Tori	13	Climber	Fruit	Helps in curing anemia and liver disorders	Nutrient supplement	Cultivated	0.081
*Momordica charantia* L.	GUH-21029	Karela	3	Climber	Fruit	Helpful in curing diabetes	Nutrient supplement	Commonly cultivated	0.019
Dipterocarpaceae									
*Shorea robusta* Gaertn.	GUH-21030	Sal	6	Tree	Bark	Bark paste is used in treating skin diseases.	None	Common	0.037
Discoreaceae									
*Dioscorea bulbifera* L.	GUH-21031	Genthi	3	Climber	Tuber	Cooked tubers are used in curing ulcers and piles.	Tuber used as vegetable	Common	0.019
Ericaceae									
*Rhododendron arboreum* Sm.	GUH-21032	Burans	5	Tree	Flower	Flower juice is used for curing blood pressure.	Leaves used for livestock fodder	Common	0.031
Euphorbiaceae									
*Phyllanthus emblica* L.	GUH-21033	Amla	33	Tree	Fruit	Hair wash, skin smoothening.	Nutrient supplement	Common	0.205
*Sapium insigne* (Royle) Benth. & Hook. f.	GUH-21034	Khinnu	9	Tree	Leaf	Leaf paste is used on burns.	Used for ritual purposes.	Common	0.056
Fagaceae									
*Quercus leucotrichophora* A. Camus	GUH-21041	Banj	5	Tree	Gum	Gum of plant obtained is boiled with small amount of cow urine and then applied on the areas around broken horn.	For livestock fodder, doors, windows and fuelwood	Abundant	0.031
Juglandaceae									
*Juglans regia* L.	GUH-21042	Akhrot	12	Tree	Fruit, Root, Twig	Root bark and twig are used for khurpakka treatment. Fruit peel is useful for the treatment of ringworm.	Tooth cleaning	Common	0.075
Lamiaceae									
*Mentha arvensis* L.	GUH-21043	Pudina	7	Herb	Whole plant	Stomach ache treatment.	As condiment	Uncommon	0.043
*Ocimum sanctum* L.	GUH-21044	Ban tulsi	14	Herb	Whole plant	Diabetes, cough treatment.	For ritual purpose	Commonly cultivated	0.087
*Perilla frutescens* (L.) Britton	GUH-21045	Bhangzeera	7	Herb	Leaf	Juice extracted after crushing leaf is used in earache.	Nutrient supplement	Common	0.043
*Vitex negundo* L.	GUH-21046	Siwali	21	Shrub	Leaf, stem	Paste of leaf is used for treating wounds, paralysis and rheumatic pain. Stem paste is used to control fever.	Used for ritual purposes	Common	0.13
Lauraceae									
*Cinnamon tamala* Nees & Eberm.	GUH-21047	Tejpatta	26	Tree	Stem, Bark, Leaf	Stem and bark is used in curing cough. Leave is used in curing throat, asthma problems.	As flavouring agent in dishes.	Common	0.161
Leguminosae									
*Bauhinia vahlii* Wight. &Arn.	GUH-21048	Malu	11	Climber	Root	Pyorrhea treatment	For livestock fodder	Abundant	0.086
*Bauhinia variegate* L.	GUH-21049	Kweral	11	Tree	Leaf, Bark	Leaf paste in skin disease, bark powder in treating blood pressure	Nutrient supplement, livestock fodder, firewood	Common	0.068
*Abrus precatorius* L.	GUH-21035	Ratti	3	Climber	Root	Root is used to cure ulcer and rheumatic pain	None	Common	0.019
*Butea monosperma* (Lam.) Taub.	GUH-21036	Dhak	3	Tree	Seed, Flower	Seed and flower are used in the treatment of dysentery and ringworm.	None	Common	0.019
*Glycine max* (L.) Merr.	GUH-21037	Kala bhatt	11	Herb	Seed	Seed paste is used for eye sores.	Nutrient supplement	Cultivated	0.068
*Macrotyloma uniflorum* (Lam.) Verdc.	GUH-21038	Ghaith	26	Herb	Seed	Seed soaked in water are helpful in treating kidney stones	Nutrient supplement	Commonly cultivated	0.161
*Ougeinia oojeiensis* Hochr.	GUH-21039	Sandhan	2	Tree	Gum	Gum is used for treating digestive trouble.	Wood for timber and firewood purpose	Common	0.012
*Trigonella foenum-graecum* L.	GUH-21040	Methi	47	Herb	Leaf, Seed	Helps in lowering cholesterol	Used as flavoring agent in Indian dishes	Often cultivated	0.292
Linaceae									
*Reinwardtia indica* Dumort.	GUH-21050	Phionly	6	Herb	Whole plant	Used for treating cattle diseases and wounds.	Used as livestock fodder	Common	0.037
Lythraceae									
*Punica granatum* L.	GUH-21051	Anar	13	Tree	Root	Roots are grinded to powder and then mixed with half liter of water and fed to animal to remove internal parasite.	Nutrient supplement	Cultivated	0.081
*Woodfordia fruiticosa* L.	GUH-21052	Dhaula	2	Shrub	Flower	Dried flowers are used in dysentery.	Used for livestock fodder	Common	0.012
Malvaceae									
*Bombax ceiba* L.	GUH-21053	Semal	5	Tree	Bark	Curing joint break	Nutrient supplement	Common	0.031
*Grewia optiva* Drumm. ex Burret	GUH-21086	Bhimal	27	Tree	Bark, Twig, Leaf	Hair wash	Rope making, fuelwood, fodder	Common	0.037
Meliaceae									
*Melia azedarach* L.	GUH-21054	Dainkan	7	Tree	Leaf, Seed, Bark, Root	Treating skin disease	For livestock fodder.	Common	0.043
Menispermaceae									
*Tinospora cordifolia* (Willd.) Miers ex Hook. f. & Thomson	GUH-21055	Gilloi	31	Climber	Twig	Fever, respiratory problems, indigestion treatment.	None	Not uncommon	0.193
Moraceae									
*Ficus subincisa* Buch.-Ham. ex Sm.	GUH-21056	Umaru	6	Tree	Latex	Latex with boiled water is used for treating diarrhea, piles and diabetes.	For livestock fodder	Common	0.037
*Ficus roxburghii* Wall.	GUH-21057	Timla	35	Tree	Latex, Fruit	Latex is used to cure cuts and wound. Roasted fruit is used to cure diarrhea and dysentery.	Nutrient supplement, fuelwood, fodder	Common	0.217
*Ficus palmate* Browicz	GUH-21058	Bedu	10	Tree	Latex	Skin wound	Nutrient supplement	Common	0.062
*Morus serrata* L.	GUH-21059	Shahtoot	9	Tree	Fruit	Curing anemia	Fodder, basket making, fuelwood, nutrient supplement	Not uncommon	0.056
Musaceae									
*Musa paradisiaca* L.	GUH-21060	Banana	19	Herb	Fruit	In curing high blood pressure, asthama, daibetes.	Nutrient supplement	Cultivated	0.118
Myricaceae									
*Myrica esculenta* Buch.-Ham. ex D. Don	GUH-21061	Kaphal	12	Tree	Fruit, Bark	Fruit eaten when mixed with mustard oil and salt is helpful in digestion. Stem bark powder is used for cough.	Fruit is used as nutrient supplement	Abundant	0.075
Myrtaceae									
*Psidium guajava* L.	GUH-21062	Guava	11	Tree	Leave	Curing cough.	Nutrient supplement	Cultivated	0.068
*Syzygium cumini* (L.) Skeels	GUH-21063	Jamun	4	Tree	Fruit	Helps in treating diabetes	Nutrient supplement	Common	0.025
Oxalidaceae									
*Oxalis corniculata* L.	GUH-21064	Khatibuti/Tipati	10	Herb	Leaf	Leaf paste is applied on skin ulcer and wound.	Nutrient supplement	Common	0.062
Pedaliaceae									
*Sesamum orientale* L.	GUH-21065	Til	23	Herb	Seed	Curing joint pain	For ritual purpose	Common	0.143
Pinaceae									
*Pinus roxburghii* Sarg.	GUH-21066	Kulah	39	Tree	Resin, Pollen	Resin is used as crack cream. Pollen dust and resin with water is used for cancer and tuberculosis treatment.	Wood for timber and firewood, needles are used for livestock bedding and for ritual purpose	Abundant	0.242
Poaceae									
*Cynodon dactylon*(L.) Pers.	GUH-21067	Doob	18	Herb	Whole plant	Plant juice is used for fever and burning sensation.	Used in rituals	Common	0.112
*Echinochloa crusgalli* L.	GUH-21068	Jhangora	14	Herb	Seed	Curing loose motion.	Nutrient supplement	Commonly cultivated	0.087
*Hordeum vulgare* L.	GUH-21069	Jau	12	Herb	Leaf	Leaf juice is used for cataract.	Nutrient supplement and livestock fodder	Commonly cultivated	0.075
*Oryza sativa* L.	GUH-21070	Satti	13	Herb	Seed	Helpful in curing constipation.	Nutrient supplement and livestock fodder	Commonly cultivated	0.081
*Zea mays* L.	GUH-21071	Mungri	5	Herb	Seed	Flour of dry seed is used for digestion.	For livestock fodder	Commonly cultivated	0.031
Polygonaceae									
*Rumex hastatus* D. Don	GUH-21072	Almoda	5	Herb	Leaf	Leafpaste is applied on fungal infection.	Nutrient supplement	Fairly common	0.031
Rhamnaceae									
*Ziziphus mauritiana* Lam.	GUH-21073	Ber	3	Shrub	Root	Roots are used in treating human indigestion.	Nutrient supplement	Fairly common	0.019
*Ziziphus nummularia* (Burm. f.) Wight & Arn.	GUH-21074	Jharber	9	Shrub	Fruit, Leaf	Fruit as astringent, leaves for treating skin diseases.	Nutrient supplement	Common	0.056
Rosaceae									
*Prunus cerasifera* Ehrh.	GUH-21075	Poolam	28	Tree	Fruit	Fruit act as antioxidant, helps in lowering cholesterol.	Nutrient supplement, leaves for livestock	Commonly cultivated	0.174
*Prunus cerasoides* Buch.-Ham. ex D. Don	GUH-21076	Panya	39	Tree	Fruit, bark	Fruit is astringent and digestive. Juice of bark is applied externally to treat backaches.	Ritual, fuelwood, nutrient supplement	Common	0.242
*Prunus persica* (L.) Batsch.	GUH-21077	Aadu	11	Tree	Leaf	Treating khurpakka	Nutrient supplement	Commonly cultivated	0.068
*Pyrus pashia* Buch.-Ham. ex D. Don	GUH-21078	Melu	17	Tree	Fruit	Juice of ripped fruit is used to cure conjunctivitis.	Nutrient supplement, fodder, fuelwood	Fairly common	0.106
*Rosa macrophylla* Lindl.	GUH-21079	Gulaab	2	Shrub	Flower	Skin moisturizer	For ritual purpose	Uncommon	0.012
*Rubus ellipticus* Sm.	GUH-21080	Hisoola	5	Shrub	Root	Root paste is applied on ulcer and skin infection.	Nutrient supplement	Common	0.031
Rutaceae									
*Aegle marmelos* (L.) Correa	GUH-21081	Bel	5	Tree	Fruit	Fruit is used for diabetes treatment.	Leave is used for ritual purpose	Common	0.031
*Citrus aurantifolia* Swingle	GUH-21082	Nimbu	5	Tree	Fruit	Diarrhea treatment	Nutrient supplement	Uncommon	0.031
*Murraya koenigii* (L.) Spreng.	GUH-21083	Kadipatta	3	Shrub	Leave	Respiratory problem	As flavoring agent in dishes	Common	0.019
*Zanthoxylum armatum* DC.	GUH-21084	Timru	23	Shrub	Bark, Fruit, Twig	Toothache treatment	Tooth cleaning	Common	0.143
Solanaceae									
*Datura stramonium* L.	GUH-21085	Dhatura	15	Herb	Leaf, Flower	Leave and flower is used to cure bronchitis, asthma and cough.	Fruit is used for ritual purpose	Common	0.168
Urticaceae									
*Urtica dioica* L.	GUH-21087	Kandali	6	Herb	Leaf	Stomach-ache	Rope making, nutrient supplement	Common	0.106
Zingiberaceae									
*Cautleyaspicata* (Sm.) Baker	GUH-21088	Jadhaldu	17	Herb	Rhizome	Rhizome paste useful in treatment of skin burns.	None	Common	0.093
GUH-Garhwal University Herbarium.									
